# Co-sleeping with pets, stress, and sleep in a nationally-representative sample of United States adults

**DOI:** 10.1038/s41598-024-56055-9

**Published:** 2024-03-06

**Authors:** Brian N. Chin, Tvisha Singh, Aisha S. Carothers

**Affiliations:** Department of Psychology, Trinity College, 300 Summit Street, Hartford, CT 06106 USA

**Keywords:** Psychology, Human behaviour, Risk factors

## Abstract

This cross-sectional study tested the direct and stress-buffering effects of co-sleeping with pets on human sleep characteristics in a nationally-representative sample of United States adults. Participants completed questionnaires assessing their sleep characteristics, including perceived sleep quality, perceived sleep efficiency, insomnia severity, and multidimensional sleep health. We evaluated whether co-sleeping with pets was associated with sleep characteristics and whether co-sleeping with pets moderated the association of stress and sleep characteristics. Exploratory analyses examined whether sleep characteristics were impacted by number of pets, pet type, and bondedness to pets. Our final sample of 1591 participants (*M*_*age*_ = 46.4 years, *SD* = 17.5; 56% female; 76% White) included 758 participants who reported co-sleeping with pets (47.6%). Co-sleeping with pets was associated with poorer sleep characteristics—specifically, poorer perceived sleep quality and greater insomnia severity. Although higher levels of stress were associated with poorer sleep, we did not observe evidence for a stress-buffering effect of co-sleeping with pets. Exploratory analyses indicated that the negative impact of co-sleeping with pets on human sleep was associated with dog ownership but not cat ownership, more pronounced when individuals own a greater number of pets, and not impacted by bondedness to pets. Our findings contribute to emerging evidence for the impact of co-sleeping with pets on human sleep. Study was pre-registered at: https://aspredicted.org/3VN_WF6.

## Introduction

Human-animal relationships are ubiquitous in modern society. In fact, it is estimated that two-thirds of households in the United States include at least one pet animal^1^. Pets are a recognized social determinant of health that have been shown to reduce disease risk and promote health and well-being, in part, because of their influence on humans’ daily behavioral routines^2^, including sleep–wake patterns^3^. However, despite the prevalence of human-animal cohabitation in modern society, the influence of pets on human sleep remains understudied relative to the impact of co-sleeping with other humans, such as one’s spouse or children^4^.

Much like the effects of co-sleeping with other humans, there are theorized benefits and drawbacks of co-sleeping with pets for humans’ sleep characteristics^3,5^. The beneficial effects of co-sleeping with pets on human sleep are usually attributed to the human-pet relationship conferring a sense of psychological security, comfort, and intimacy^6,7^ that may promote better sleep by reducing nighttime cognitive arousal^8^. In theory, these benefits may be especially evident for individuals who are currently experiencing high levels of psychological stress (i.e., a stress-buffering effect of co-sleeping with pets). Moreover, pets may also function as social zeitgebers that facilitate more regular and robust circadian rhythms, in part, by establishing daily feeding and exercise routines^9,10^. Conversely, the potential drawbacks of co-sleeping with pets on human sleep are mostly attributed to pets serving as a source of nighttime noise, heat, and/or movement which could disturb humans’ ability to maintain continuous and deep sleep throughout the night^11^. Indeed, the nature and timing of how most pets sleep (including both dogs and cats) tends to be misaligned with humans’ diurnal and monophasic sleep patterns^5^. It is therefore unsurprising that more than half of pet owners report being woken up nightly by their pets^3^.

Some earlier studies have found that co-sleeping with pets may negatively impact humans’ sleep^3,5^. For example, an investigation of 10,128 Australian adults by Smith et al.^12^ found that participants who reported co-sleeping with pets took longer to fall asleep at night, were more likely to report feeling tired upon waking, and were more likely to report noise-related nighttime sleep disturbances than an age- and gender-matched sample of individuals who did not co-sleep with pets. However, co-sleeping with pets was not associated with average sleep duration or daytime tiredness in this investigation. Similar results were found in a small study of 40 dog owners by Patel et al.^13^, who reported that dog owners had poorer actigraphy-assessed sleep efficiency when their dogs slept in the bed as opposed to just being in the room.

In contrast, other studies have found that co-sleeping with pets may have a neutral or mixed impact on humans’ sleep. A study of 962 adult women in the United States by Hoffman et al.^11^ reported that dog and cat owners who co-slept with their pets did not differ from non-owners on any subscale of the Pittsburgh Sleep Quality Index except for daytime dysfunction. Another study of 1356 Australian participants by Hoffman et al.^14^ also found no overall impact of co-sleeping with dogs on owners’ self-reported sleep quality. Moreover, this study found that owners who did not co-sleep with their dogs were more likely to report frequently waking up tired than owners who did co-sleep with their dogs; it was hypothesized that their dog’s presence could provide owners with a sense of comfort and security that facilitates relaxation and sleep. Consistent with this proposed explanation, participants in this study were more likely to report bedsharing with their dog when they perceived greater emotional closeness to their dog and reported more frequent interactions with their dog.

Thus, there remains a need for additional evidence regarding the effect of co-sleeping with pets on sleep quality and other sleep characteristics, including assessments of insomnia symptom severity^15^ and multidimensional sleep health^16^. Such evidence would be helpful for evaluating the veracity of claims regarding the problematic impact of pets in the bedroom^17^ and for elucidating why human-animal co-sleeping practices continue to remain commonplace in modern societies despite their apparent drawbacks^3^. Moreover, there remains a need for tests of the potential stress-buffering effect of co-sleeping with pets on sleep characteristics—that is, the possibility that pets may help to protect humans against the negative effects of stress on sleep^18^. To our knowledge, earlier studies have exclusively assessed the direct effects of pets on sleep, and none have statistically tested for possible stress-buffering effects. However, there is considerable evidence from qualitative studies of pets serving a stress-buffering function for humans’ sleep by reducing bedtime physiological and cognitive arousal^19^ and by serving as a source of reassurance, comfort, and distraction from nighttime anxiety and worry^10,20^. In theory, these psychological benefits of co-sleeping with pets may help humans to initiate and maintain sleep more easily even when they are feeling stressed or overwhelmed.

Finally, there is a need to understand whether specific pet characteristics are related to humans’ sleep characteristics, including number pets, pet type, and degree of bondedness to pets. These characteristics have been underexamined in earlier studies of pets and sleep. First, few previous studies have examined whether number of pets impacts sleep characteristics, although Hoffman et al.^14^ reported that the number of dogs in the house was not associated with humans’ sleep quality or likelihood of frequently waking up tired. Second, most prior studies in this area have either not assessed pet type^12^ or focused on dogs only^13,14^. However, one study that examined both dog and cat owners found that participants who co-slept with dogs reported greater comfort and security than those who co-slept with cats or humans and less nighttime disturbance than those who co-slept with humans^11^. However, this study did not find significant differences in any subscale of the Pittsburgh Sleep Quality Index according to pet type. Third, we are unaware of any prior studies that have tested whether bondedness to pets is associated with humans’ sleep characteristics. Although Hoffman et al.^14^ reported that bedsharing with dogs was more common for individuals who reported greater emotional closeness to their pet, they did not report whether emotional closeness was associated with sleep quality.

### Objectives

This study tested the direct and stress-buffering effects of co-sleeping with pets on human sleep characteristics in a nationally-representative sample of United States adults. First, we hypothesized that we would observe a direct effect of co-sleeping with pets on sleep characteristics; however, given the mixed prior evidence regarding the impact of co-sleeping with pets on humans’ sleep characteristics, we did not make a specific hypothesis regarding the nature of this effect. Second, we hypothesized that we would observe a stress-buffering effect of co-sleeping with pets on humans’ sleep characteristics. Specifically, we hypothesized that co-sleeping with pets would protect humans from the negative effects of stress on sleep characteristics (i.e., that higher stress would be less harmful to the sleep of individuals who are co-sleeping with pets). Finally, we tested the exploratory aims of whether type of pet, number of pets, or bondedness to pets was associated with humans’ sleep characteristics.

## Method

### Participants and procedures

Participants were recruited for a cross-sectional observational study of pets, stress, and sleep habits. We determined our target sample size by conducting an a priori power analysis using G*Power^21^ to determine the sample size needed to test our first aim. Our power analysis tested the number of participants needed to detect group differences in sleep characteristics using an independent means t-test with two tails and an error probability of ɑ = 0.05, desired power of 0.95, and estimated allocation ratio of 1. The required total sample size to detect a small between-group difference (*d* = 0.2) was *N* = 1300. Study inclusion criteria were being ≥ 18 years old and fluent in English. There were no exclusionary criteria. Study procedures were approved by the Trinity College Institutional Review Board. All methods were carried out in accordance with relevant guidelines and regulations.

We recruited participants through Prime Panels^22^, an online survey recruitment platform that aggregates opt-in market research panels to enable data collection based on demographic quotas. Specifically, we aimed to recruit a sample of 1300 adults who were representative of the United States population with regard to age, sex, race, and ethnicity based on US Census demographic distributions. After providing informed consent, participants completed a Qualtrics questionnaire assessing their pet ownership, sleep characteristics, perceived stress, and demographic characteristics. Participants with pets also completed additional questionnaires assessing their sleeping arrangement and the strength of their bond to their pets. Participants received financial compensation for completing the study that depended on the platform used to access the survey. Data were collected on August 2, 2023; recruitment was halted in 9.5 h after all demographic quotas had been reached.

### Measures

#### Co-sleeping with pets

Participants were asked about their current pet ownership (yes/no). Pets were defined for participants as “the animals that we keep in and around our homes for companionship, including dogs, cats, birds, reptiles, fish, etc.” Participants who responded yes to this item were asked to indicate where they slept in relation to their pets (not in the same room, in the same room but apart, in the same room and together). Participants were categorized as *co-sleeping with pets* if they responded that they slept in the same room with their pet either together or apart, or *not co-sleeping with pets* if they reported not owning a pet or owning a pet but not sleeping in the same room.

#### Pet characteristics

Current pet owners also completed an additional questionnaire assessing characteristics of themselves and their pets. This questionnaire included items assessing their current number of pets (one, between two and five, between six and ten, and more than 10), type(s) of pets (dog, cat, fish, bird, reptile, rabbit, hamster, guinea pig, ferret, horse, and other), and whether the participant served as the primary caregiver for at least one of their pets (yes/no). This questionnaire also included the 7-item Pet Bondedness Scale^23^ which assesses how strongly an individual is bonded with their pets (e.g., “I tell others that my pet(s) are a member of my family.”). Items were rated on a five-point scale ranging from 1 (strongly disagree) to 5 (strongly agree) with a neutral midpoint of 3 (neither agree nor disagree). This measure provides an overall human-pet bondedness score that ranges from 7 to 35 with higher scores representing greater human-pet bondedness. Participants completed two items assessing the overall perceived impact of their pets on their sleep and on their health and wellness were rated on a five-point scale from 1 (very negative) to 5 (very positive) with a midpoint of 3 (neutral).

#### Sleep characteristics

Participants completed questionnaires assessing their sleep characteristics. Sleep quality was assessed using the 10-item Pittsburgh Sleep Quality Index^24^ which asks participants to rate their sleep habits during the past month on the seven dimensions of subjective quality, sleep latency, sleep duration, habitual sleep efficiency, sleep disturbances, daytime alertness, and use of sleep medication. Consistent with earlier investigations^25,26^, we conducted an exploratory factor analysis of the seven components of the Pittsburgh Sleep Quality Index to identify the latent factor structure in this sample. Insomnia severity was assessed using the 7-item Insomnia Severity Index^15^ which asks participants to rate the extent to which they have experienced insomnia problems during the past two weeks. This measure provides an overall insomnia severity score that is calculated by summing the item scores. Insomnia severity scores range from 0 to 28 with higher scores representing greater insomnia severity. Multidimensional sleep health was assessed using the 6-item R-SATED questionnaire^16^ which asks participants to rate their general sleep habits. This measure provides an overall multidimensional sleep health score that is calculated by summing the item scores for sleep regularity, satisfaction, alertness, timing, efficiency, and duration. Multidimensional sleep health scores range from 0 to 12 with higher scores representing better sleep health.

#### Perceived stress

Participants completed the 10-item version of the Perceived Stress Scale^27^ assessing the extent to which their life was unpredictable, uncontrollable, and overwhelming during the past month. Items were rated on a five-point scale from 1 (never) to 5 (very often). This measure provides an overall perceived stress score that is calculated by summing the item scores. Perceived stress scores range from 10 to 50 with higher scores representing greater perceived stress.

#### Demographic characteristics

Participants completed a demographic questionnaire assessing their age (continuous), sex (categorical: female, male, did not disclose), race/ethnicity (categorical: White, Black, Hispanic or Latino, Asian or Asian American, American Indian or Alaska Native, Native Hawaiian or Pacific Islander), income (ordinal: less than $35,000, $35,000 to $75,000, $75,000 to $150,000, more than $150,000), and educational attainment (ordinal: less than high school, high school or equivalent, some college and no degree, associate’s degree, bachelor’s degree, graduate degree).

### Data analysis

We tested Aim 1 by conducting a one-way multivariate analysis of variance (MANOVA) with perceived sleep quality, perceived sleep efficiency, insomnia severity, and multidimensional sleep health as the dependent variables, age and sex as covariates, and co-sleeping with pets as the independent variable. Pairwise comparisons were used to evaluate the significance of between-group differences in sleep characteristics based on the estimated marginal means and standard errors.

We tested Aim 2 by conducting a two-way MANOVA with perceived sleep quality, perceived sleep efficiency, insomnia severity, and multidimensional sleep health as the dependent variables, age and sex as covariates, and co-sleeping with pets, perceived stress, and the interaction of co-sleeping with pets x perceived stress as the independent variables.

We tested our first exploratory aim by conducting a one-way MANOVA with perceived sleep quality, perceived sleep efficiency, insomnia severity, and multidimensional sleep health as the dependent variables, age and sex as covariates, and co-sleeping dog owner (yes/no) and co-sleeping cat owner (yes/no) as the independent variables.

We tested our second and third exploratory aims by conducting a one-way MANOVA on the subsample of participants who co-sleep with pets (*n* = 758) with perceived sleep quality, perceived sleep efficiency, insomnia severity, and multidimensional sleep health as the dependent variables, age and sex as covariates, and number of pets and bondedness to pets as the independent variables.

### Transparency and openness

Our study was pre-registered with AsPredicted prior to conducting this research at: https://aspredicted.org/3VN_WF6; we did not pre-register our specific hypotheses or exploratory analyses. We also note the following deviations from the pre-registration: (1) we pivoted our data analytic strategy to a MANOVA to reduce the likelihood of Type I error; and (2) our final sample was larger than planned because additional participants were recruited to meet demographic quotas. We conducted our analyses using SPSS software (Version 29.0, IBM, Armonk, NY, USA) using the GLM command. We prepared this manuscript according to the STROBE Statement recommendations for reports of cross-sectional studies.

## Results

### Descriptive results

Our final sample consisted of 1591 participants who ranged in age from 18 to 91 years. Most participants identified as female (56.1% female, 43.5% male, 0.4% did not disclose) and White (75.5% White, 12.8% Black, 10.8% Hispanic or Latino, 4.1% Asian, 2.8% American Indian or Alaska Native, 0.4% Native Hawaiian or Pacific Islander). Most participants had not completed a college degree (54.3%) and reported an annual household income of less than $75,000 (70.1%). We examined the number of participants who used a response set to answer the sleep characteristic and perceived stress measures as a means of estimating the rate of bot-generated data or inattentive responding in this sample. There were seven participants (0.4%) who used a response set to answer all four of these questionnaires. Because similar results were obtained when including and excluding these participants from our analyses, we elected to retain these individuals in the final analyses reported below.

Table 1 summarizes the demographic and sleep characteristics of the full sample and compares the characteristics of participants who did and did not co-sleep with pets. Our sample included 758 participants who co-sleep with pets (47.6%) and 833 participants who did not co-sleep with pets (52.4%). Participants who co-slept with pets were younger and more likely to be female and white compared to those who did not co-sleep with pets. These groups did not differ in their income or educational attainment.

**Table 1:** Demographic and sleep characteristics of the full sample and stratified by group.

	Full sample (*N* = 1591)	Co-sleeps with pets (*N* = 758)	Does not co-sleep with pets (*N* = 833)	*p* diff
Age, years	46.4 (17.5)	44.2 (16.1)	48.4 (18.5)	< 0.001
Sex, % (*n*)				0.010
Female	56.1 (892)	60.0 (455)	52.5 (437)	
Male	43.5 (692)	39.6 (300)	47.1 (392)	
Did not disclose	0.4 (7)	0.4 (3)	0.5 (4)	
Race, % (*n*)				< 0.001
White	75.5 (1201)	81.4 (617)	70.1 (584)	
Black	12.8 (203)	8.2 (62)	16.9 (141)	
Hispanic or Latino	10.8 (172)	11.7 (89)	10.0 (83)	
Asian or Asian American	4.1 (66)	1.8 (14)	6.2 (52)	
American Indian or Alaska Native	2.8 (45)	2.2 (17)	3.4 (28)	
Native Hawaiian or Pacific Islander	0.4 (6)	0.5 (4)	0.2 (2)	
Educational attainment, % (*n*)				0.71
Less than high school	2.5 (39)	2.6 (20)	2.3 (19)	
High school or equivalent	27.4 (436)	28.2 (214)	26.7 (222)	
Some college; no degree	24.5 (389)	23.9 (181)	25.0 (208)	
Associate’s degree	11.9 (189)	12.4 (94)	11.4 (95)	
Bachelor’s degree	22.1 (351)	19.9 (151)	24.0 (200)	
Graduate degree	11.8 (187)	12.9 (98)	10.7 (89)	
Income, % (*n*)				0.72
Less than $35,000	34.4 (547)	33.6 (255)	35.1 (292)	
$35,000 to $75,000	35.8 (569)	36.9 (280)	34.7 (289)	
$75,000 to $150,000	22.3 (354)	21.2 (161)	23.2 (193)	
Over $150,000	7.6 (121)	8.2 (62)	7.1 (59)	
Sleep characteristics, *M(SD)*				
Perceived sleep quality	0.0 (1.0)	0.2 (1.0)	− 0.2 (1.0)	< 0.001
Perceived sleep efficiency	0.0 (1.0)	0.0 (1.0)	− 0.0 (1.0)	0.24
Insomnia severity	9.6 (6.4)	10.6 (6.4)	8.8 (6.2)	< 0.001
Multidimensional sleep health	7.6 (2.7)	7.4 (2.5)	7.7 (2.8)	0.017

*Note.* Age, educational attainment, income, and sleep characteristic differences were evaluated using an independent-samples *t*-test with equal variances not assumed. Sex and race differences were evaluated using chi-squared tests.

Sleep characteristic scores ranged from 0 to 28 for insomnia severity (*M* = 9.6, *SD* = 6.4) and from 0 to 12 for multidimensional sleep health (*M* = 7.6, *SD* = 2.7). Insomnia severity and multidimensional sleep health scores were normally distributed with skewness values of 0.5 and − 0.2 and kurtosis values of − 0.4 and − 0.4. As shown in Table 2, the exploratory factor analysis of the seven components of the Pittsburgh Sleep Quality Index produced a two-factor solution in this sample. We extracted one factor that consisted of the subjective sleep quality, sleep latency, sleep disturbances, sleep medication use, and daytime dysfunction subscales that was labeled perceived sleep quality, and a second factor that consisted of the sleep duration and habitual sleep efficiency subscales that was labeled perceived sleep efficiency. Perceived sleep quality (*M* = 0.00, *SD* = 1.00) and perceived sleep efficiency scores (*M* = 0.00, *SD* = 1.00) were normally distributed with skewness values of 0.3 and 0.7 and kurtosis values of − 0.4 and − 0.4. The bivariate correlations of sleep characteristic variables are shown in Table 3.

**Table 2:** Factor matrix for the two-factor solution of the Pittsburgh Sleep Quality Index.

PSQI Subscale	Factor 1: perceived sleep quality	Factor 2: perceived sleep efficiency
Subjective sleep quality	0.63	0.23
Sleep latency	0.61	0.25
Sleep duration	0.12	0.78
Habitual sleep efficiency	− 0.04	0.84
Sleep disturbances	0.74	0.05
Sleep medication use	0.60	− 0.23
Daytime dysfunction	0.74	− 0.02
% of total variance	37.73	16.75

**Table 3:** Bivariate correlations of sleep characteristic variables in the full sample (*N* = 1591).

	1	2	3	4
1. Perceived Sleep Quality	–			
2. Perceived Sleep Efficiency	0.22	–		
3. Insomnia Severity	0.76	0.39	–	
4. Multidimensional Sleep Health	− 0.40	− 0.42	− 0.52	–

Note. All correlations were statistically significant at *p* < 0.001.

#### Pet characteristics

Among the 758 participants who reported co-sleeping with their pets, all owned either dogs (76.0%) or cats (54.1%); fewer also owned fish (5.4%), birds (2.8%), reptiles (3.8%), rabbits (1.6%), hamsters (0.7%), guinea pigs (0.9%), ferrets (0.3%), horses (0.7%), or other types of pets (2.2%). Nearly all participants reported having either one pet (39.6%) or between 2 and 5 pets (52.2%) and functioning as the primary caregiver for at least one of their pets (92.0%). Most rated the overall impact of their pets on their sleep as neutral (33.9%), positive (33.2%), or very positive (26.4%) with fewer rating this impact as negative (5.7%) or very negative (0.8%). Similarly, most rated the overall impact of their pets on their health and wellness as positive (42.5%) or very positive (40.1%) with fewer rating this impact as neutral (15.3%), negative (1.7%), or very negative (0.4%).

### Aim 1: association of co-sleeping with pets and sleep characteristics

We observed a statistically significant difference in the combined sleep dependent variable between participants who did and did not co-sleep with pets, *F*(4,1584) = 8.13, *p* < 0.001, Wilks’ Λ = 0.980, partial η^2^ = 0.020. Results of the follow-up univariate tests suggested that this was attributable to between-group differences in perceived sleep quality, *F*(1,1587) = 29.28, *p* < 0.001, partial η^2^ = 0.018, and insomnia severity, *F*(1,1587) = 21.39, *p* < 0.001, partial η^2^ = 0.013, but not perceived sleep efficiency, *F*(1,1587) = 0.36, *p* = 0.55, partial η^2^ < 0.001, or multidimensional sleep health, *F*(1,1587) = 0.96, *p* = 0.33, partial η^2^ = 0.001.

**Figure 1:**
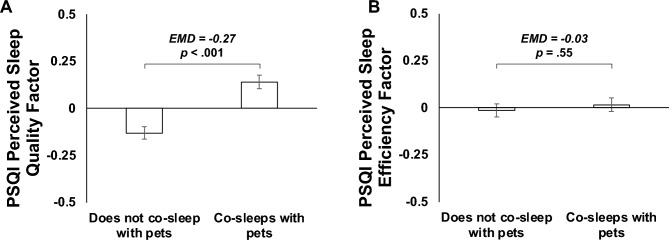
Plots illustrating the covariate-adjusted estimated mean differences (EMD) in the perceived sleep quality (Panel **A**) and perceived sleep efficiency (Panel **B**) of participants who did and did not co-sleep with pets.

**Figure 2:**
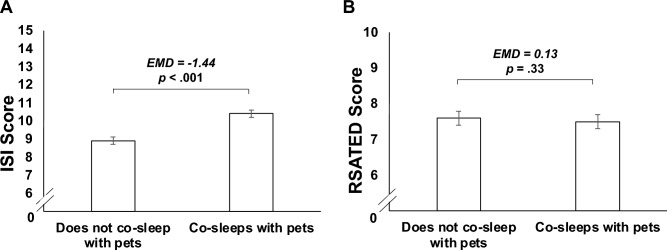
Plots illustrating the covariate-adjusted estimated mean differences (EMD) in the insomnia severity (Panel **A**) and multidimensional sleep health (Panel **B**) of participants who did and did not co-sleep with pets.

As shown in Figs. 1A and 2A, participants who reported co-sleeping with pets had poorer perceived sleep quality (*EMD* = − 0.27, *SE* = 0.05, *p* < 0.001, *95CI* = − 0.36, − 0.17) and greater insomnia severity (*EMD* = − 1.44, *SE* = 0.31, *p* < 0.001, *95CI* = − 2.06, − 0.83) than those who did not co-sleep with pets. As shown in Figs. 1B and 2B, these groups did not differ in their perceived sleep efficiency (*EMD* = − 0.03, *SE* = 0.05, *p* = 0.55, *95CI* = − 0.13, 0.07) or multidimensional sleep health (*EMD* = 0.13, *SE* = 0.13, *p* = 0.33, *95CI* = − 0.13, 0.38).

### Aim 2: stress-buffering effects of co-sleeping with pets on sleep characteristics

There was a statistically significant association of perceived stress and the combined sleep dependent variable, *F*(4,1582) = 155.49, *p* < 0.001, partial η^2^ = 0.282. Results of the follow-up univariate tests suggested that this was attributable to associations with worse perceived sleep quality, *F*(1,1585) = 506.16, *p* < 0.001, partial η^2^ = 0.242, worse perceived sleep efficiency, *F*(1,1585) = 54.16, *p* < 0.001, partial η^2^ = 0.033, greater insomnia severity, *F*(1,1585) = 486.00, *p* < 0.001, partial η^2^ = 0.235, and worse multidimensional sleep health, *F*(1,1585) = 184.17, *p* < 0.001, partial η^2^ = 0.104.

Contrary to our hypothesis, we did not observe a statistically significant interaction of co-sleeping with pets × perceived stress on the combined sleep dependent variable, *F*(4,1582) = 0.38, *p* = 0.82, Wilks’ Λ = 0.999, partial η^2^ = 0.001.

### Exploratory aim 1: analyses of pet type

There were statistically significant differences in the combined sleep dependent variable between participants who did and did not co-sleep with dogs, *F*(4,1583) = 5.63, *p* < 0.001, partial η^2^ = 0.014, and who did and did not co-sleep with cats, *F*(4,1583) = 2.69, *p* = 0.030, partial η^2^ = 0.007.

For co-sleeping with dogs, results of the follow-up univariate test suggested that this was attributable to between-group differences in perceived sleep quality, *F*(1,1586) = 18.82, *p* < 0.001, partial η^2^ = 0.012, perceived sleep efficiency, *F*(1,1586) = 4.21, *p* = 0.039, partial η^2^ = 0.003, and insomnia severity, *F*(1,1586) = 18.75, *p* < 0.001, partial η^2^ = 0.012, but not multidimensional sleep health, *F*(1,1586) = 2.75, *p* = 0.10, partial η^2^ = 0.002. Participants who co-slept with dogs reported poorer perceived sleep quality (*EMD* = − 0.23, *SE* = 0.05, *p* < 0.001, *95CI* = − 0.34, − 0.13), poorer perceived sleep efficiency (*EMD* = − 0.11, *SE* = 0.05, *p* = 0.039, *95CI* = − 0.22, − 0.01), and greater insomnia severity (*EMD* = − 1.46, *SE* = 0.34, *p* < 0.001, *95CI* = − 2.12, − 0.80) than those who did not co-sleep with dogs. These groups did not differ in their multidimensional sleep health (*EMD* = 0.23, *SE* = 0.14, *p* = 0.10, *95CI* = − 0.04, 0.51).

For co-sleeping with cats, results of the follow-up univariate test suggested that this was attributable to between-group differences in perceived sleep efficiency only, *F*(1,1586) = 5.07, *p* = 0.024, partial η^2^ = 0.003. We did not observe between-group differences in perceived sleep quality, *F*(1,1586) = 3.26, *p* = 0.07, partial η^2^ = 0.002, insomnia severity, *F*(1,1586) = 1.02, *p* = 0.31, partial η^2^ = 0.001, or multidimensional sleep health, *F*(1,1586) = 0.18, *p* = 0.67, partial η^2^ < 0.001. Participants who co-slept with cats reported *better* perceived sleep efficiency (*EMD* = 0.13, *SE* = 0.06, *p* = 0.024, *95CI* = 0.02, 0.25) than those who did not co-sleep with cats. These groups did not differ in their perceived sleep quality (*EMD* = − 0.11, *SE* = 0.06, *p* = 0.07, *95CI* = − 0.22, 0.01), insomnia severity (*EMD* = − 0.37, *SE* = 0.37, *p* < 0.31, *95CI* = − 1.10, 0.35), or multidimensional sleep health (*EMD* = − 0.07, *SE* = 0.15, *p* = 0.67, *95CI* = − 0.37, 0.24).

### Exploratory aims 2–3: analyses of number of pets and bondedness

We observed a statistically significant difference in the combined sleep dependent variable based on the number of pets owned, *F*(4,750) = 2.56, *p* = 0.037, Wilks’ Λ = 0.987, partial η^2^ = 0.013, but not bondedness to pets, *F*(4,750) = 1.41, *p* = 0.23, Wilks’ Λ = 0.993, partial η^2^ = 0.007. Results of the follow-up univariate tests suggested that this was attributable to an association of number of pets with greater insomnia severity, *F*(1,753) = 3.88, *p* = 0.049, partial η^2^ = 0.005. Number of pets was not associated with perceived sleep quality, *F*(1,753) = 3.43, *p* = 0.06, partial η^2^ = 0.005, perceived sleep efficiency, *F*(1,753) = 1.01, *p* = 0.32, partial η^2^ = 0.001, or multidimensional sleep health, *F*(1,753) = 0.50, *p* = 0.48, partial η^2^ = 0.001.

## Discussion

This study evaluated the direct and stress-buffering effects of co-sleeping with pets on sleep characteristics in a nationally-representative sample of American adults. First, we found evidence for direct effects of co-sleeping with pets on sleep characteristics. Participants who reported co-sleeping with pets had poorer perceived sleep quality and greater insomnia severity than those who did not; however, co-sleeping with pets was not associated with perceived sleep efficiency or multidimensional sleep health. Second, we did not find evidence for stress-buffering effects of co-sleeping with pets on sleep characteristics. Although participants with higher levels of psychological stress reported poorer sleep across all characteristics, there was no evidence that co-sleeping with pets was protective against this negative impact. Our exploratory analyses also indicated that (1) the direct effect of co-sleeping with pets on sleep characteristics may depend on the type of pet owned with more consistent evidence for a disrupting effect of co-sleeping with dogs than cats; (2) having a greater number of pets was associated with greater insomnia severity (but not other sleep characteristics); and (3) bondedness to pets was not associated with any sleep outcome among participants who co-slept with pets. Overall, our study contributes to an emerging literature on pets and sleep by providing additional evidence for a negative overall impact of co-sleeping with pets on humans’ sleep characteristics that may depend on both the number and type of pets.

### Direct and stress-buffering effects of co-sleeping with pets

Co-sleeping with pets was associated with poorer sleep characteristics—specifically, poorer perceived sleep quality and greater insomnia severity. This observation is consistent with the findings of an earlier investigation by Smith et al.^12^ which found that adults who co-slept with pets took longer to fall asleep, were more likely to feel tired upon waking, and were more likely to report disturbances due to animal-related noises at night than those who did not co-sleep with pets. Our results extend this earlier finding by indicating that co-sleeping with pets may specifically impact the dimensions of perceived sleep quality and insomnia severity but not multidimensional sleep health or perceived sleep efficiency. These observations contrast with the findings of an earlier investigation of American women by Hoffman et al.^11^ which found no impact of dog or cat ownership on the PSQI global score or any of its individual components except for daytime dysfunction. Possible explanations for these discrepant findings are that our investigation had a larger control group than Hoffman et al.^11^ and that we scored the PSQI using a factor analytic approach instead of a global score. Each of these differences may have increased the statistical power to detect a between-group difference in our study. In particular, earlier investigations have found that the PSQI global score has questionable psychometric properties and suggested that the multifaceted nature of sleep may be better captured by a scoring approach that is multidimensional rather than unidimensional^25,26^.

It is notable that co-sleeping with pets did not predict poorer sleep across all outcomes. This observed mixed impact is consistent with a recent narrative review that found preliminary evidence for a negative effect of co-sleeping with pets on humans’ sleep, but hypothesized that some of these deficits may be counteracted by the psychological benefits of bedsharing with pets^5^. Notably, we found evidence for harmful direct effects of co-sleeping with pets on perceived sleep quality and insomnia severity but not multidimensional sleep health or perceived sleep efficiency. We speculate that this pattern may be attributable to the different dimensions of sleep that are captured by these assessments. Our measures of perceived sleep quality and insomnia severity, but not perceived sleep efficiency or multidimensional sleep health, included items assessing sleep disturbances and sleep problems (e.g., difficulty falling or staying asleep). This is consistent with the possibility that the negative effects of co-sleeping with pets on sleep are attributable to pets serving as a source of nighttime disturbance. Future studies should continue to evaluate nighttime disturbances due to movement, noise, or heat as a primary candidate mechanisms by which co-sleeping with pets impacts human sleep.

This study also provided an initial test of the hypothesized stress-buffering effect of co-sleeping with pets on humans’ sleep. Contrary to our prediction, we did not observe a stress-buffering effect of co-sleeping with pets on sleep characteristics. Although higher levels of perceived stress were associated with poorer sleep, we did not find evidence that co-sleeping with pets was protective against this negative impact. This was surprising given that pet owners qualitatively report that co-sleeping with pets can provide a source of reassurance, comfort, and distraction from bedtime stress and anxiety^10,19,20^. We hypothesize that a reason we did not observe stress-buffering effects of co-sleeping with pets in this study is because our assessments of perceived stress and sleep characteristics asked participants to summarize these variables across the past weeks or month. It is possible that the hypothesized stress-buffering effects may occur on a shorter timescale than we captured in this study, such as if co-sleeping with pets was protective against decrements in sleep on the night(s) that follow a particularly stressful day. Future studies could test this hypothesis by using daily diaries or ecological momentary assessments to measure stress and sleep at a finer temporal resolution. Another possible reason that we did not observe stress-buffering effects of co-sleeping with pets is because we examined a general sample that was intended to be representative of the United States population, whereas earlier studies have documented qualitative evidence for stress-buffering effects of pets in more specific populations, such as individuals with chronic pain^10,20^ and veterans with posttraumatic stress disorder^19^. Finally, it is possible that we did not observe evidence for a stress-buffering effect of co-sleeping with pets because our exposure variable compared individuals who co-sleep with pets to those who do not. Thus, our comparison group also included some individuals who did not co-sleep with their pets but who may still have benefitted from the stress-reducing effects of interacting with their pets during the daytime.

The results of our exploratory aims suggested several novel hypotheses about how specific pet characteristics impact human sleep that should be tested in subsequent studies. First, we found stronger evidence for a negative impact of dogs than cats on human sleep. When considering the type of pets owned by individuals who co-sleep with pets, we found that dog ownership was associated with poorer sleep characteristics across all outcomes besides multidimensional sleep health, whereas cat ownership predicted *better* perceived sleep efficiency and was not associated with other sleep characteristics. This is a notable contribution to the literature because most prior studies of pets and sleep have not assessed pet type^12^ or focused on dogs only^13,14^. However, this finding is also somewhat surprising given that an earlier study comparing the impact of cats and dogs on sleep found that humans perceived co-sleeping with dogs to provide more comfort and security than co-sleeping with cats^11^. It is possible that the observed harmful effect of co-sleeping with dogs on human sleep characteristics in this study could be explained by dogs serving as a source of nighttime movement that disturbs sleep. Consistent with this explanation, initial studies of people who co-sleep with dogs have found that greater nighttime dog movement and activity is associated with more sleep disturbances and worse sleep quality for humans^28,29^. Given the exploratory nature of our findings, the impact of pet type on humans’ sleep should continue to be explored in subsequent studies.

Second, we found that having a greater number of pets was associated with greater insomnia severity. Our observation contrasts with the results of Hoffman et al.^14^ who found that the number of dogs in the house or bed was not associated with humans’ sleep quality or likelihood of waking up tired. One possible explanation for this difference is that our study assessed the presence of other animals in the home besides dogs that can also serve as a source of nighttime sleep disturbance due to noise or movement. However, few previous studies have examined whether number of pets impacts humans’ sleep and future research is needed to determine whether this association replicates in other samples. In addition, our assessment of pet number was relatively crude as nearly all participants who co-slept with pets reported having either one pet or between two and five pets.

Third, we did not find evidence that humans’ degree of bondedness to their pets impacted their sleep characteristics. This was surprising given that we had expected humans who were more bonded to their pets to derive the greatest psychological benefits (i.e., comfort, security) from co-sleeping. Because Hoffman et al.^14^ found that dog owners were more likely to bedshare with their dog when they felt greater emotional closeness to their pet, it is possible that there was a restricted range of bondedness among those co-sleeping with pets in our study which limited our ability to detect an association with sleep characteristics. Alternatively, it is possible that the impact of co-sleeping with pets on human sleep characteristics could depend on other qualities of the pet-human bond, such as the nature of humans’ attachment to their pets^3^.

### Strengths and limitations

Strengths of this study include its pre-registration, adherence to transparency and openness guidelines, assessment of multiple sleep characteristics, and examination of a nationally-representative sample of adults in the United States. In addition, there are several limitations of this study that provide opportunity for future investigation.

First, our study’s cross-sectional design limits the extent to which we can infer causal links between co-sleeping with pets, stress, and sleep. We acknowledge the possibilities of reverse-causation (i.e., that poor sleepers are more likely to have and co-sleep with pets) and third-factor explanations. Important third factors that we did not assess in this study include the impact of other humans on sleep (e.g., spouse or children), the role of other social zeitgebers like employment or school schedules, the use of sleep medications, and the presence of a sleep disorder or other psychological or medical condition that may impact sleep and the likelihood of having pets. Subsequent research should address this limitation by using longitudinal study designs that can provide evidence for the temporal nature of these associations. It is particularly important for future studies to assess whether individuals also co-sleep with other humans given that earlier research has found that human–dog bedsharing was less likely for those who slept with another human in the bed^14^ and that bedsharing with other humans also impacts sleep^5^.

Second, our study’s questionnaire-based assessment of sleep characteristics may have been susceptible to the limitations and cognitive biases associated with self-report measures. This limitation is especially salient given that studies of human bedsharing have found divergent impacts on subjective and objective measures of sleep^5^. Future studies should address this limitation by evaluating the impact of co-sleeping with pets on objective measures of sleep. Of note, several initial studies have already used accelerometer data to study sleep in smaller samples of dog owners^13,28,29^.

Third, our study data were collected via panel service and online survey which may have resulted in the inclusion of both inattentive participants and bot participants. We did not employ attention checks or any similar technique to eliminate such participants. As such, this could have introduced additional error variability into our analyses that increased the likelihood of Type-II error. However, our analyses suggested that there was a minimal amount of bot participants and inattentive participants in this sample.

### Constraints on generality

We also acknowledge several constraints on the external validity of our findings. The main boundary condition of this research is its examination of adults from the United States. It is unknown whether similar effects of co-sleeping with pets would be observed in other societal and cultural contexts. Although pets are common around the world, there is significant variation in co-sleeping practices and the extent to which pets are regarded as members of one’s family both within and between cultures that may subsequently influence how pets impact humans’ sleep^30–32^. For example, Volsche^33^ has suggested that pet parenting may be more common in cultures that have experienced the second demographic transition away from the norms of conventional parenthood. Thus, it would be important for future studies to assess the possible role of pet and human parenting in promoting or deterring human-pet co-sleeping practices. For example, Volsche et al.^34^ found that co-sleeping with pets was more common for participants who self-identified as non-parents (i.e., those who did not have or plan to have children) than those who were current parents or who intended to become parents in the future.

Because we did not assess the health status of our participants, a second boundary condition is that it is unclear whether these results can be generalized to individuals living with disabilities or chronic illness. It is similarly unclear whether these effects would be observed among individuals with allergies who may experience greater sleep disturbances when co-sleeping with their pets due to increased allergen exposure^35^.

A third boundary condition of this work is that we assessed the association of co-sleeping with pets and sleep for adults only. Thus, it is unclear whether these results are generalizable to the many children and teenagers who live and co-sleep with pets. For example, one recent study of adolescents found that co-sleeping with pets was not associated with self-reported sleep quality^36^.

## Conclusions

Human-animal relationships are common and on the rise in the United States. Our findings add to emerging evidence that human sleep may be disturbed when co-sleeping with pets, especially dogs. Because good sleep is essential for maintaining optimal health and well-being, our results suggest a need for continued exploration of the impact of co-sleeping with pets on humans’ sleep. This work would support public health efforts to promote sleep at the population level and help to progress scientific sleep research by expanding the focus from the individual to include the broader environment—including their animal companions.

## Data Availability

Our dataset and analytic code are publicly available in the OSF repository at: https://osf.io/afn9x/?view_only=94bedeb776874222aac8d04009bf8e5c.
